# Pain Care Management in Rare Diseases

**DOI:** 10.3390/healthcare11192628

**Published:** 2023-09-27

**Authors:** Emérito Carlos Rodríguez-Merchán, Matteo Nicola Dario Di Minno, Gianluigi Pasta

**Affiliations:** 1 Department of Orthopedic Surgery, La Paz University Hospital, 28046 Madrid, Spain; 2 Osteoarticular Surgery Research, Hospital La Paz Institute for Health Research—IdiPAZ La Paz University Hospital—Autonomous University of Madrid, 28046 Madrid, Spain; 3 Department of Translational Medical Sciences, Federico II University, 80131 Napoli, Italy; dario.diminno@hotmail.it; 4 Department of Orthopedic Surgery, IRCCS Fondazione Policlinico San Matteo, 27100 Pavia, Italy; gianluigipasta@yahoo.it

In this Special Issue on “Musculoskeletal Pain Care and Management in Rare Disease”, it is essential to make it clear that, while specialists in rare diseases (RDs) are often very knowledgeable about the management of the specific diseases in which they are experts, primary care physicians and other physicians who are not experts in a given disease often have very little contact with the patients who experience it. Therefore, they often know little about the treatment of a particular RD and the management of pain associated with it [1]. Fortunately, there are many websites that allow physicians and patients to acquire information about RDs, and it is quite common for patients to know more about their own disease than the non-specialist physicians who treat them [2].

On the other hand, it can take many years for a given RD to be detected because of previous misdiagnoses that delay specialty care [3]. Such delayed diagnoses can have very negative consequences, sometimes causing the disease to progress uncontrollably and increase in severity, especially in terms of persistent and worsening pain. Regardless of pain management, the treatment of RDs is a great challenge due to their different etiologies, pathophysiologies and anatomical areas affected. Therefore, in this setting, it is necessary to have a multidisciplinary team (i.e., with members from the fields of orthopedic surgery, pharmacy, hematology, immunology, physical and rehabilitation medicine, neurology, psychiatry, pain expertise, etc.) [4].

Chronic pain, in many RDs, may begin in childhood or become more noticeable in adulthood, and it often affects the quality of life of the patients who experience them [5]. Therefore, the fundamental goal in RDs is to use effective symptomatic or curative treatments [6]. The 2020 publication by The Lancet commission on pediatric pain recommended trials in children with RDs with the aim of preventing long-term pain [7]. It is important to remember that there are approximately 7000 currently known RDs.

With regard to chronic childhood pain in RDs, Sieberg et al. have stated that living with an RD can be terrible, not only for the children who experience them, but also for their families and for society in general. This is because multiple and continuous surgical interventions are often necessary and because RDs cause disability, rejection and acute and/or chronic pain due to pathophysiological or iatrogenic causes (i.e., induced by the treatments themselves that have to be performed throughout their lives). It is therefore essential to provide children with psychosocial care regarding their pain. There is also a need to increase the awareness of physicians, scientists and policy makers to this important medical and public health need that has not yet been sufficiently covered. The assessment of chronic pain in children with RDs should be performed using a multidisciplinary and interdisciplinary approach based on a team formed by pediatricians and experts in pain medicine, mental health, surgery, anesthesiology, endocrinology, social services and community outreach specialists [8].

Regarding pediatric pain, a recent publication has stated that a considerable percentage of parents hold misconceptions about how children express pain, and therefore, we must encourage the formation of programs for parents to help identify, assess and correctly manage pain in their children [9]. According to Collins (2023), in children with severe pain and disability, intensive interdisciplinary pain management might be required to improve pain and function [10]. Heydinger et al. (2023) have reported that regional anesthesia and virtual reality are valuable tools that serve to relieve pain in children [11]. Lalloo et al. have recently published an article that shows that Project ECHO^®^ (Project Extension for Community Healthcare Outcomes) is a feasible model for the virtual education of interprofessional healthcare providers in treating pediatric pain [12].

With regard to chronic pain in adults with RDs affecting the musculoskeletal system, in 2020, Mickute et al. published the most important priorities to be considered in the management of adult patients with rare bone metabolic disorders [13] (Table 1).

According to Tucker-Bartley et al., pain frequently occurs in rare musculoskeletal and neuromuscular diseases. Moreover, pain mechanisms, phenotypes and therapeutic modalities often overlap significantly. On the other hand, there is a paucity of controlled clinical trials evaluating pain in RDs, which often leads to inappropriate or ineffective pain management. Future research should study the efficacy of specific or disease-modifying medications aimed at pain management, as pain reduces quality of life and even functional status in some patients [14].

In 2022, Gutenbrunner et al. demonstrated that RD patients experience functional restrictions in the areas of mental well-being and activity. As a result, health-related quality of life decreases compared to healthy individuals. Almost half of patients studied experienced significant pain-related impairments, although only 9% of them had received adequate pain therapy [15].

According to Delande and Lavand’homme (2023), efficacious acute pain treatment is paramount but is currently suboptimal, which could impact long-term results. Optimal multimodal analgesia to spare opioid administration is given priority, as opioids might enhance neuroinflammation, which underlies pain persistence and precipitates neurocognitive decline in frail individuals. In addition, recent findings have shown that acute pain treatments which modulate nociceptive and inflammatory pain should be utilized with caution, as medications which inhibit inflammation like NSAIDS and corticosteroids may interfere with natural recovery processes [16].

In conclusion, RDs often present acute and/or chronic pain which must be adequately treated. To this end, it is very important to have good knowledge of the clinical and scientific basis for their management. Furthermore, we believe that we must increase the awareness and sensitivity of clinicians and scientists to this important medical and public health need, which unfortunately has not yet been sufficiently covered. Therefore, there is no doubt that pain management in patients with RDs must be improved, for which multidisciplinary work is essential. We hope that this Special Issue can help all physicians to remember the importance of pain in patients with RDs and its correct management.

## Figures and Tables

**Table 1:** Research priorities for rare bone metabolic disorders (RBMDs) in adults [13].

TREATMENT	What is considered a good outcome for a given treatment? How can a good outcome be assessed in studies of new treatments? What is the best way to treat fatigue associated with RBMDs? What are the advantages and disadvantages of the medications used to manage patients with RBMDs in the short and long term, and what is the optimal duration of these medications? What are the best surgical interventions to treat the bone and joint lesions suffered by patients with RBMDs?
PROGNOSIS	What is the cause of pain in patients with RBMDs? How do RBMDs worsen with age and how do they differ from the aging that takes place in healthy people? How and why do people with RBMDs have different symptoms even if they have the same genetic mutation?
SUPPORT AND CARE	What is the psychological impact of suffering from RBMD and how can patients and their families best be helped?
PREVENTION	How can RBMDs be prevented or prevented from worsening?
SELG-MANAGEMENT	What is the best way to prevent dental problems in people with RBMDs?
